# Advanced CRISPR-Cas Effector Enzyme-Based Diagnostics for Infectious Diseases, Including COVID-19

**DOI:** 10.3390/life11121356

**Published:** 2021-12-07

**Authors:** Sangha Kwon, Ha Youn Shin

**Affiliations:** Department of Biomedical Science and Engineering, Konkuk University, Seoul 05029, Korea; tkdgk2219@naver.com

**Keywords:** CRISPR-CAS, Cas12, Cas13, molecular diagnosis, COVID-19, SARS-CoV-2

## Abstract

Rapid and precise diagnostic tests can prevent the spread of diseases, including worldwide pandemics. Current commonly used diagnostic methods include nucleic-acid-amplification-based detection methods and immunoassays. These techniques, however, have several drawbacks in diagnosis time, accuracy, and cost. Nucleic acid amplification methods are sensitive but time-consuming, whereas immunoassays are more rapid but relatively insensitive. Recently developed CRISPR-based nucleic acid detection methods have been found to compensate for these limitations. In particular, the unique collateral enzymatic activities of Cas12 and Cas13 have dramatically reduced the diagnosis times and costs, while improving diagnostic accuracy and sensitivity. This review provides a comprehensive description of the distinct enzymatic features of Cas12 and Cas13 and their applications in the development of molecular diagnostic platforms for pathogen detection. Moreover, it describes the current utilization of CRISPR-Cas-based diagnostic techniques to identify SARS-CoV-2 infection, as well as recent progress in the development of CRISPR-Cas-based detection strategies for various infectious diseases. These findings provide insights into designing effective molecular diagnostic platforms for potential pandemics.

## 1. Introduction

Recent global pandemics have highlighted the importance of accurate diagnostic tests for infectious and pathogenic diseases. Immunoassays for infectious diseases are widely used to detect pathogen-associated antigens or antibodies against pathogens in patient serum. Although these assays are relatively simple to perform and require a short turnaround time, they are relatively insensitive, making it difficult to evaluate clinical samples containing low concentrations of target molecules [1,2,3]. Moreover, because several days are needed for patients to make sufficient concentrations of antigens or antibodies to be detected by these assays, they are not suitable for early diagnosis. Recently developed immunoassay-based rapid diagnostic kits only take about 15 min to perform and are suitable for point-of-care diagnosis, but their low sensitivity yields high percentages of false-negative and false-positive results [4,5,6]. Because immunoassays are relatively inaccurate, nucleic-acid-amplification-based tests have become the standard method for the early diagnosis of coronavirus disease 2019 (COVID-19). These tests use polymerase chain reaction (PCR) to amplify small amounts of nucleic acid sequences specific to pathogens, making them extremely sensitive and highly specific for the target molecules [7]. However, several hours (4–6 h) are required to obtain the test results. Moreover, PCR-based methods require expensive reagents, special equipment, and well-trained personnel who can accurately perform these analyses. These limitations may be overcome by developing more advanced diagnostic tools that are rapid, cost-effective, and sensitive.

Among the new diagnostic approaches that have been developed recently are those involving clustered regularly interspaced short palindromic repeats (CRISPR) [8,9,10]. CRISPR and CRISPR-associated proteins (Cas) were originally detected as components of the adaptive immune systems in bacteria and archaea, defending these organisms against invading viruses by degrading foreign genetic elements [11,12]. The CRISPR-Cas system consists of two major components, a guide RNA (gRNA) and Cas endonuclease. The gRNA is made up of two parts: crispr RNA (crRNA), a 17–20 nucleotide sequence complementary to the target RNA or DNA, and a trans-activating CRISPR RNA (tracrRNA), which serves as a binding site for the Cas nuclease [13,14]. Following the binding of crRNA and tracrRNA to a complementary foreign sequence, the Cas nuclease recognizes the target for cleavage and induces double-strand breaks (DSB) in the genome [15]. The CRISPR-Cas system has been classified into two classes and six types of Cas proteins [16,17,18]. Class 1 systems (types I, III, and IV) rely on multi-protein effector complexes, whereas class 2 systems (types II, V, and VI) function via a single effector protein.

Of the various types of Cas nucleases identified to date, Cas9 has been the most well studied and is commonly used for genome editing. Bacterial CRISPR-Cas9 was re-engineered into a simple two-component system by combining crRNA and tracrRNA into a single-guide RNA (sgRNA) [19]. This re-engineering simplified the manipulation of the nucleotide sequence of gRNA to target any genomic region. This artificial CRISPR-Cas9 system revolutionized genome editing, leading to the wide applicability of this system to editing genes of various organisms. Because of its ability to edit genes and regulate transcription, Cas9 endonuclease has also been used diagnostically [20,21,22,23]. CRISPR-based diagnostic (CRISPR-Dx) uses a Cas9 mutant that has lost its cleavage activity, retaining only its DNA-binding ability [24]. This Cas9 mutant can be further linked to a reporter system, such as a fluorescent tag. Thus, the Cas9 mutant can recognize binary complexes formed by the binding of a gRNA to a specific pathogenic sequence, with the linked reporter system activated only if the pathogens are present in clinical samples.

Following the initial use of Cas9 in CRISPR-based diagnostic platforms, different Cas enzymes have been used to improve CRISPR-Dx. For example, Cas12 and Cas13 were each shown to have a unique enzymatic activity that cleaves the target nucleotide sequence followed by the cleavage of neighboring non-target sequences [9,10,25]. This collateral cleavage activity was employed as a “switch” to turn on various types of reporter molecules [19,26,27,28]. In addition, Cas12 and Cas13 are more flexible in selecting target molecules and target sequences. Unlike Cas9, which recognizes only double-stranded DNA (dsDNA), Cas12 can target both dsDNA and single-stranded DNA (ssDNA) and Cas13 can target RNA molecules (Table 1). Therefore, all types of DNA and RNA viruses can be targeted using CRISPR-Dx. Cas9 typically cleaves the three nucleotides upstream of the protospacer active motif (PAM), consisting of the sequence NGG (where N represents any nucleotide) within a target region. Because Cas12 and Cas13 recognize different target sequences, they provide more opportunities to select appropriate target regions. Moreover, Cas12a, one of the Cas12 enzymes, and Cas13 do not require tracrRNA, needing only crRNA for their activity. Due to this distinct feature, it is simple and more convenient to design gRNAs that target pathogenic sequences.

This review describes the unique features of Cas12 and Cas13 that play pivotal roles in CRISPR-Cas-based diagnostics. Recent CRISPR-Cas-based diagnostic platforms for molecular diagnosis of various infectious diseases, including COVID-19, are comprehensively described. This review will assist researchers in understanding the advantages and limitations of currently developed CRISPR-Cas-based diagnostic methods and may aid in the development of advanced diagnostic strategies for future epidemics.

## 2. Molecular Diagnosis Based on the CRISPR Effectors Cas12 and Cas13

### 2.1. Cas12-Based Diagnostics

The Cas12 enzymes are type V RNA-guided DNA-targeting enzymes, consisting of several subtypes [18]. Among these subtypes are Cas12a (formerly Cpf1), Cas12b (previously C2C1), and Cas12f (also known as Cas14), all of which have been frequently used in CRISPR-Cas-based diagnostics to detect infectious viruses and bacteria. Cas12 cleaves target DNA by recognizing T-rich PAM sequences (TTN), generating staggered 5′ overhangs. Intriguingly, Cas12 has collateral or trans-cleavage activity, enabling it to recognize target sequences while cleaving adjacent non-target sequences [8,26]. Cas12a and Cas12b can target ssDNA as well as dsDNA [25,29]. Unlike Cas9 and Cas12b, which require both crRNA and tracrRNA, Cas12a only uses crRNA, making it relatively easier to design a gRNA [25,26,29]. Cas12f, which is about half the size (400–700 aa) of Cas9 or Cas12 (950–1400 aa), was originally categorized as the type V effector protein Cas14, based on its targeting of ssDNA independent of PAM sequences [10,30]. More recent studies have shown, however, that Cas14 effector is similar to Cas12, as it can also target dsDNA, dependent on T-rich PAM [31]. These results, together with mining of sequence databases, resulted in the re-classification of Cas14 into the Cas12 family [32,33].

In contrast to Cas9, the more versatile features of Cas12, especially its trans-cleavage activity, make it more suitable for molecular diagnoses [8,26]. These functions can be used to activate various types of reporter molecules. Based on these properties, Cas12-based diagnostic platforms have been developed by coupling several types of reporter systems, including a fluorescent probe, a lateral flow readout, and an electrochemical sensor (Table 2).

#### 2.1.1. Fluorescence-Based Methods

**Figure 1:**
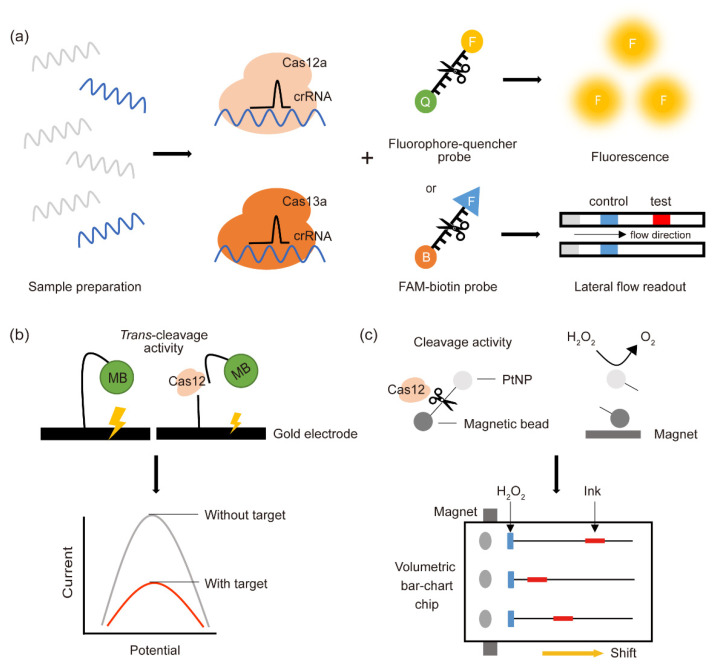
Schematic illustration of CRISPR-Cas-based diagnostic platforms using Cas12 and Cas13. (**a**) CRISPR-Cas-based detection methods coupled with a fluorescent probe (upper panel) or a lateral flow readout (lower panel). CRISPR-Cas variants recognize the target sequences of pathogenic DNA and RNA, collaterally cleaving the ssDNA or ssRNA linked to the fluorophore-quencher or FAM-biotin reporter. Released fluorescent probes are captured and quantified by a fluorescence detection instrument, whereas released FAM molecules are visualized on paper strips. (**b**) CRISPR-Cas-based electrochemical biosensor (E-CRISPR) with a methylene blue (MB) probe. Upon CRISPR-Cas12 recognition of the target sequence, Cas12 collaterally cleaves the ssDNA linker of the MB electrochemical tag, reducing the electric current. (**c**) Combination of a magnet-assisted volumetric bar-chart chip (MAV-chip), a platinum nanoparticle (PtNP), and CRISPR-Cas12a. In the presence of target DNA, Cas12 cleaves the target sequence as well as the adjacent ssDNA reporter between the PtNP and a magnetic bead. The cleaved PtNP converts H_2_O_2_ to O_2_, with the produced O_2_ gas shifting the ink a distance proportional to the amount of target DNA.

The first Cas12-based diagnostic method was developed in 2018 by coupling Cas12a with a fluorophore quencher (FQ)-labeled reporter molecule (Figure 1a, top panel) [8]. Formation of a ternary complex of CRISPR-Cas12a with the target DNA was found to induce the cleavage by Cas12a of either dsDNA or the ssDNA FQ reporter through its trans-cleavage activity, activating the fluorescent probe. This technique resulted in the successful and specific detection of two dsDNA viruses, human papillomavirus types 16 (HPV16) and 18 (HPV18), within 1 h. To further improve the diagnostic sensitivity of this assay, the target DNA was amplified by recombinase polymerase amplification (RPA) under isothermal conditions (37 °C for 10 min), a technique called the DNA endonuclease targeted CRISPR trans-reporter (DETECTR) method. Using this approach, DETECTR was able to accurately detect HPV16 and HPV18 in clinical samples with attomolar (aM, 10^−18^ M) sensitivity. DETECTR clearly offered an advanced molecular diagnostic strategy that is rapid, specific, and sensitive, although it could not distinguish between targets differing by a single nucleotide.

The target specificity of Cas12a-based diagnostic tests was further improved by PCR amplification of the target DNA rather than single-temperature amplification [34]. This technique, called a one-hour low-cost multipurpose highly efficient system (HOLMES), was able to detect Pseudorabies virus (PRV) and Japanese encephalitis virus (JEV) with a detection sensitivity as low as 1–10 aM. In addition, HOLMES was able to distinguish between virus strains and between nucleic acids differing by a single nucleotide. Despite its higher specificity and sensitivity, HOLMES requires a longer time than other CRISPR-Cas-based diagnostic tools to obtain test results, primarily because the PCR amplification takes at least 45 min. This drawback was overcome in 2019 by the development of a more advanced form of HOLMES, called HOLMESv2, by adopting a method called loop-mediated isothermal amplification (LAMP) [35]. LAMP is an emerging amplification technique that amplifies multiple targets (usually 4–6 sites) at a constant temperature (typically 60–65 °C) using several different primer sets [46]. An additional pair of “loop primers” can further enhance the specificity and efficacy of the reaction. Due to the simultaneous amplification of multiple targets at constant temperature, LAMP is more rapid and can produce a 100-fold higher number of DNA copies than conventional PCR. HOLMESv2 used the enzyme Cas12b instead of Cas12a. Like Cas12a, Cas12b can recognize both dsDNA with T-rich PAM and ssDNA independent of PAM. Coupling of Cas12b to LAMP is sufficiently sensitive to distinguish between targets differing by a single nucleotide and enables the detection of the RNA virus JEV in 1 h using reverse transcriptase (RT)-LAMP.

Another Cas12b-based diagnostic tool, called Cas12b-mediated DNA detection (CDetection), was developed in 2019 [29]. Compared with the Cas12a-based detection method, CDetection exhibited higher sensitivity with low background signals and could distinguish between HPV16 and HPV18. When coupled with RPA, CDetection showed a 1 aM level of detection sensitivity on HPV dsDNA in human plasma, indicating that a single drop of blood is sufficient to detect infectious viruses. In addition, CDetection was able to distinguish among cancer-related single-nucleotide mutations (TP53 856G>A, BRCA1 3232A>G and 3537A>G) by coupling with a tuned guide RNA (tgRNA), which introduces single-nucleotide mismatches in the spacer for reducing the off-target effect [47]. CDetection achieved the lowest detection sensitivity among Cas12-based diagnostic methods, with a limit of detection (LoD) of 0.1 aM.

CRISPR-*Mycobacterium tuberculosis* complex (CRISPR-MTB), which was developed as a diagnostic method to detect *M. tuberculosis* [36], showed greater sensitivity than culture or GeneXpert MTB/RIF (Cepheid Inc., Sunnyvale, CA, USA) assays. In addition, CRISPR-MTB could be used to detect *M. tuberculosis* in various clinical samples, including sputum, bronchoalveolar lavage fluid (BALF), and cerebrospinal fluid (CSF).

Cas12f (formerly known as Cas14), which is half the size of the other Cas12 enzymes, has collateral activity at non-specific nucleotides while recognizing its target, enabling its use in CRISPR-based diagnoses. Based on this collateral activity, the combination of CRISPR-Cas14a and the fluorescent reporter (Cas14a-DETECTR) was found to successfully target the specific single-nucleotide polymorphism (SNP) responsible for eye color, an SNP not detected using Cas12a [10]. Although Cas14a-DETECTR is not yet widely used in pathogen identification and the detailed underlying mechanism of Cas12f remains to be determined, Cas12f could have great potential in diagnosing pathogens, similar to Cas12a and Cas12b.

#### 2.1.2. Lateral Flow Readout

Although fluorescence-based diagnostic methods are quick and extremely sensitive, they are not suitable for on-site detection, due to the requirement for expensive fluorescence detection equipment. This issue was solved by the development of a lateral flow visual readout platform using a FAM-biotin reporter [43]. Lateral flow detection assays use the binding affinities of various molecules, such as antigen–antibody or biotin–streptavidin binding. Pathogens targeted by gRNAs and FAM-biotinylated ssDNA-linked Cas12 enzyme complexes were loaded onto a paper strip containing nanoparticles conjugated to rabbit anti-FAM antibody (Figure 1a, bottom panel). Streptavidin captures the biotin-labeled ssDNA on the control line, allowing only the Cas12-cleaved ssDNA to flow toward the test line. Cleaved-ssDNA-linked FAM–nanoparticle complexes are captured on the test line by the secondary anti-rabbit antibody, with a color change at the test line indicating a positive result. This strategy led to the development of several CRISPR-Cas12-based lateral flow assays. The Cas12a-on-site and rapid detection system (CORDS) and CRISPR-Cas12a technology and lateral flow detection (CRISPR-Cas12a-LFD) are simple, robust, and instrument-free detection platforms that have been developed for on-site detection of African swine fever virus (ASFV) [37,40]. Because both methods amplify target molecules by recombinase-aided amplification (RAA), they need only a 37~39 °C incubator and tests take only 1 h. Nevertheless, these tests are sufficiently sensitive and specific, not cross-reacting with other virus strains. In addition, CORDS is more suitable for transportation and storage in the field because the reagents can be freeze-dried, making them stable at 4 °C for at least 7 days [40]. CRISPR-Cas12a-LFD greatly reduced the detection time by removing the DNA extraction step from the pretreatment of clinical serum samples [37]. CRISPR-Cas and loop-mediated isothermal amplification (CIA) is an emerging diagnostic platform for the detection of *Pseudomonas aeruginosa* (*P. aeruginosa*) using lateral flow readout [38]. *P. aeruginosa* is an opportunistic human pathogen with multidrug resistance [48]. CIA detects the pathogen sensitively and reduces the turnaround time by omitting manual DNA extraction steps. The combination of lateral flow readout methods and a Cas effector provides field-deployable, rapid, and sensitive platforms for pathogen detection.

#### 2.1.3. Electrochemistry-Based Methods

An electrochemistry-based platform, involving an affordable transduction element and a disposable sensor, has been widely used for pathogen detection [49]. Integration of a CRISPR-Cas12-based targeting system and an electrochemical sensor further improved the accuracy of target detection. A recently developed CRISPR-Cas12a-based electrochemical biosensing platform (E-CRISPR) has shown great potential as a cheap, portable, and accurate diagnostic tool [41]. This electrochemical method combines CRISPR-Cas12a with methylene blue (MB)-tethered disposable micro-fabricated gold electrodes (Figure 1b). In the presence of a target molecule, Cas12a recognizes the target sequence and further cleaves the non-target ssDNA reporter that links MB to an electrode sensor. Consequently, MB-cleaved electrodes transduce lower electrical signals. Owing to the use of the electrochemical sensor, E-CRISPR does not require an enzymatic amplification step or its associated reagents. Using this approach, E-CRISPR was able to detect picomolar concentrations of HPV16 and nanomolar concentrations of parvovirus B19 (PB-19). In addition to detecting nucleic acids, E-CRISPR was able to detect target proteins by using an ssDNA aptamer. This method could detect transforming growth factor beta 1 (TGF-β1), a secreted protein involved in cell proliferation as well as a biomarker of hepatocellular carcinoma.

Although a paper-based visual readout is easy and deployable, it is unsuitable for quantification at the point of care [37,43]. This limitation was overcome by integrating the platinum nanoreporter-based CRISPR-Cas12a system onto a magnet-assisted V-chip (MAV-chip). This type of electrochemical method used a volumetric bar-chart channel instead of a lateral visual readout (Figure 1c) [42]. In this platform, the CRISPR-Cas12 system is coupled to platinum nanoparticles (PtNPs) tethered to magnetic beads. In the presence of target molecules, CRISPR-Cas12a binds to the target sequence, followed by the trans-cleavage of the ssDNA linkers between the PtNPs and the magnetic beads. The released PtNPs react with hydrogen peroxide to generate oxygen. The production of oxygen gas pushes the ink in the bar chart a distance proportional to the amount of the target DNA, allowing the concentration of target molecules to be quantified and visualized. This approach was capable of distinguishing single-nucleotide differences resulting from cancer-related mutations.

### 2.2. Cas13-Based Diagnostics

Cas13 is a type VI RNA-guided RNA targeting enzyme, a member of the enzyme family that includes Cas13a, Cas13b, Cas13c, and Cas13d [17,50]. Cas13a, previously called C2c2, specifically cleaves ssRNA, not dsRNA, and its unique features have been used in diagnoses [9,43]. The RNA cleavage is mediated by two higher eukaryote and prokaryote nucleotide-binding (HEPN) domains, which are commonly found in ssRNA-specific endoribonucleases, such as csm6 [28,51,52]. Similar to Cas12, Cas13 remains bound to the target region after cis on-target cleavage, further catalyzing the trans-cleavage of non-target ssRNA adjacent to the target sequence. Cas13a preferentially targets an H (non-G) protospacer flanking site (PFS), which differs from PAM of Cas9, and cleaves near uracil (U)-rich regions of ssRNA [27,28]. Further studies demonstrated the efficient RNA-targeting activity of Cas13a in mammalian cells [53], indicating that Cas13a has great potential as a diagnostic tool for detecting RNA viruses. Cas13 has been also combined with various reporter systems, including a fluorescent-tag and a lateral flow readout, to generate more versatile and robust diagnostic tools (Table 2) [9,43,44,45].

#### 2.2.1. SHERLOCK-Based CRISPR-Dx

The first Cas13a-based diagnostic platform, called the specific high-sensitivity enzymatic reporter unlocking (SHERLOCK) platform, was developed in 2017 [9]. RNA-guided RNase activity was further optimized by using an ortholog of Cas13a from *Leptotrichia wadei* (LwCas13a). Similar to Cas12a-mediated DETECTR, SHERLOCK uses a fluorescence reporter system with RPA-based target amplification. However, the biggest difference between DETECTR and SHERLOCK is that SHERLOCK requires in vitro transcription using T7 polymerase to convert amplified DNA to RNA. Despite this additional step, SHERLOCK showed attomolar sensitivity in the detection of Zika virus (ZIKV), Dengue virus (DENV), and cancer-related mutations at single-nucleotide resolution. In addition, SHERLOCK reagents are suitable for long-term storage due to lyophilization and are readily applied to paper strips for field application. Moreover, SHERLOCK only costs $0.61 per test, which is much cheaper than the usual cost of PCR or RT-PCR ($10–$15 per test) [54].

Despite its success in targeting RNA for diagnostic purposes, SHERLOCK has some limitations in the aspect of point of care. Indeed, SHERLOCK is not suitable for quantitation, and it relies on expensive fluorescence detection equipment. To overcome these limitations, more advanced versions of SHERLOCK were developed in 2018 [43,44]. SHERLOCKv2 combined multiple fluorescence reporters (e.g., FAM, TEX, Cy5, and HEX) to simultaneously detect ZIKV and DENV. By lowering the primer concentration and the amount of input samples during target preamplification, quantitative accuracy was achieved at concentrations as low as the attomolar range. The combination of Cas13 with Csm6 dramatically increased the sensitivity of detection (>3.5-fold). The use of lateral flow paper strips enabled SHERLOCKv2 to detect ZIKV and DENV within 90 min with a sensitivity as low as 2 aM and without using additional equipment.

A method called heating unextracted diagnostic samples to obliterate nucleases (HUDSON) was integrated into SHERLOCK to enable direct detection of nucleic acids in body fluid and to protect viral DNA from degradation [44]. Using heat and chemical reagents, HUDSON can disrupt viral particles and inactivate their ribonucleases, which catalyze RNA degradation. HUDSON-treated saliva or urine can be directly applied to the target amplification step of SHERLOCK. The use of HUDSON and lateral flow paper strips resulted in SHERLOCK becoming a more rapid, field-deployable diagnostic tool.

#### 2.2.2. CARMEN-Cas13

Following the development of SHERLOCKv2, the multiplexed CRISPR-Cas13-based nucleic acid detection method was further improved by integrating a high-throughput system [45]. This resulted in the development of a scalable platform harnessing microwell-array chips, called combinatorial arrayed reactions for multiplexed evaluation of nucleic acids (CARMEN). The combination of CARMEN and the CRISPR-Cas13 detection system (CARMEN-Cas13) enabled the robust detection of more than 4500 crRNA target pairs on a single array. Samples amplified by PCR or RPA on microwell plates are mixed with targeted gRNAs and fluorescent-reporter-linked Cas13 complexes. Fluorescent signals in multiple wells can therefore be simultaneously detected by fluorescence microscopy. Using this approach, CARMEN-Cas13 enabled the simultaneous detection of 169 human-associated viruses, including Middle East respiratory syndrome (MERS) virus [55] an severe acute respiratory syndrome (SARS) coronavirus 2 (SARS-CoV-2) [56], and could also differentiate among the subtypes of influenza A virus strains. CARMEN-Cas13 is highly multiplexed, sensitive (attomolar range), and much cheaper ($0.05/test) than SHERLOCKv2 ($0.61/test). The development of multiple Cas13-based diagnostic platforms, including SHERLOCK, SHERLOCKv2, HUDSON, and CARMEN-Cas13, has therefore provided new opportunities to detect viruses with susceptible RNA genomes.

## 3. Nucleic Acid Detection Platforms for SARS-CoV-2

In December 2019, a cluster of patients with atypical pneumonia caused by infection with a novel coronavirus was reported in Wuhan, China [57]. This previously unknown respiratory illness subsequently spread worldwide by rapid human-to-human transmission, resulting in an ongoing global pandemic [58]. This highly contagious acute respiratory illness was named COVID-19, and its causative agent was named severe acute respiratory syndrome coronavirus 2 (SARS-CoV-2). SARS-CoV-2 is a positive ssRNA virus that infects human cells by binding to the human receptor for angiotensin-converting enzyme 2 (ACE2) and spreads between people through close contact and via respiratory droplets. Although real-time quantitative reverse transcription PCR (RT-qPCR) is considered the standard test for the early diagnosis of COVID-19 [59,60], these tests require special equipment and trained personnel, with test results taking 4–6 h. Although immunoassays are simple and well suited for point of care testing, their accuracy is less reliable than RT-PCR methods [4,5]. Because variant mutated forms of SARS-CoV-2 keep emerging, a multiplexing, rapid, accurate, high-throughput, yet affordable diagnostic method is required to control the current pandemic. Several types of CRISPR-Cas-based diagnostic methods have therefore been developed for the molecular diagnosis of COVID-19 (Table 3).

### 3.1. Fluorescence Detection with Naked Eyes

**Figure 2:**
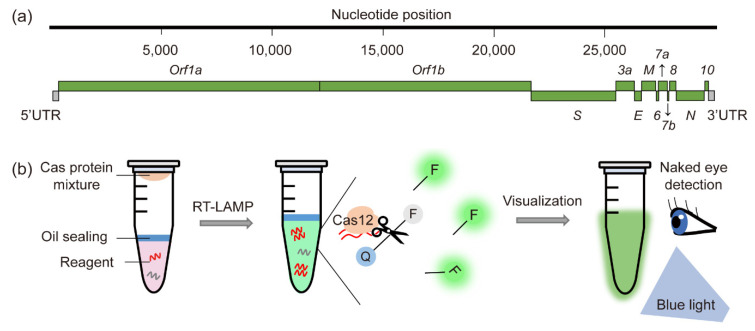
CRISPR-Cas-based detection method for SARS-CoV-2. (**a**) Genomic features of SARS-CoV-2. Typical target genes used for SARS-CoV-2 detection are those encoding the spike (S), envelope (E), and nucleocapsid (N) proteins. (**b**) Schematic illustration of the Cas12-mediated one-pot visual SARS-CoV-2 detection (opvCRISPR) method. RNA isolates and reagents for isothermal reverse amplification (RT-LAMP) are placed at the bottom the tube and sealed with oil to prevent contamination. The CRISPR-Cas12a reaction mixture is loaded inside the lid. After 40 min of RT-LAMP amplification, the tube is shaken to mix the RNA mixture and CRISPR-Cas12a reagents. Once Cas12a recognizes the target gene, the ssDNA reporter is cleaved by collateral activity. The cleaved ssDNA fluorescent reporter emits fluorescence, which can be detected under blue light with the naked eye.

CRISPR-Cas-based SARS-CoV-2 detection assays typically target the viral envelope (E), nucleoprotein (N), RNA-dependent RNA polymerase (RdRp), or spike (S) protein (Figure 2a). To inhibit the emergence of highly contagious variant SARS-CoV-2 mutants, more rapid and convenient diagnostic tools for large numbers of patient samples are required. Although CRISPR-Cas-based lateral flow strips would be one option for rapid diagnosis, a more convenient CRISPR-Cas12a-based platform, called CRISPR-Cas12a-NER, was developed, allowing the immediate confirmation of test results with the naked eye without the need for an expensive fluorescence reader [62]. In the presence of SARS-CoV-2 in clinical samples, Cas12 binds to the target sequence and cleaves the ssDNA quencher tethered to a fluorescent reporter. Released fluorescence molecules can be detected under blue light with the naked eye. Using this approach, several forms of Cas-based diagnostic platforms have been developed for SARS-CoV-2 detection, including contamination free visual detection with Cas12a [61], CRISPR-assisted detection (CASdetec) [63], a manganese-enhanced Cas12a system (MeCas12a) [64], all-in-one dual CRISPR/Cas12a assays (AIOD-CRISPR) [65], RT-RPA-CRISPR-Cas12a assays [66], RT-LAMP-Cas12a assays [67], and streamlined highlighting of infections to navigate epidemic (SHINE) [74] (Table 3). Particularly, one-pot-visual RT-LAMP-CRISPR (opvCRISPR) provided a rapid, highly sensitive, and contamination-free detection method by integrating LAMP amplification in a single tube (Figure 2b) [68]. In this method, RT-LAMP reagents are placed at the bottom of a tube and CRISPR-Cas12a reagents are added to the lid. After extracted RNA samples are mixed with the RT-LAMP reagents, RNA templates are amplified under isothermal conditions and react with CRISPR-Cas12a reagents. Cleavage of the fluorescent quencher releases fluorescence visible to the naked eye under blue light. Because all of these reactions are performed in a single tube, test results are obtained within 1 h 45 min, much faster than the traditional RT-PCR method [59]. In addition, the risk of contamination is lower, and the assays require only a thermoblock and a blue-light source, making these assays suitable for onsite diagnosis. Although the CRISPR-based fluorescent detection system (CRISPR-FDS) is more sensitive (2 copies/μL) than the opvCRISPR system (5 copies/μL), opvCRISPR is clinically more suitable and still shows much higher sensitivity than SARS-CoV-2 DETECTR (10 copies/μL) or STOPCovid.v2 (100 copies/μL) (Table 3).

### 3.2. Electrokinetic Microfluidic Chip

Although fluorescence detection with the naked eye is a user-friendly POC diagnostic tool, it is still not amenable to automation to diagnose large numbers of samples. A prerequisite RNA extraction process, which typically takes up to 1 h, also slows the test speed. These limitations may be overcome by a combination of microfluidics and an electric chip [73]. An electrokinetic microfluidic technique called isotachophoresis (ITP) was found to accelerate the speed of nucleic acid extraction from clinical samples by using a two-buffer system that migrates with different speeds on electric field gradients. A proper choice of buffers preconcentrates the target nucleic acids in ITP, while discarding any impurities. On-chip ITP further allows the automation of multiplexed CRISPR-Cas12a enzymatic activity. Consequently, the time required from acquiring nasopharyngeal swab samples to obtaining test results is only 40 min. In addition, these assays require minimal amounts of reagents, about 100-fold lower than those required for conventional methods, making the former more adaptable for automation. The specificity of this assay is similar (10 copies/µL) to that of other CRISPR-based systems. Indeed, electric-field-driven microfluidic chips provide a field deployable platform by accelerating the test speed and enabling the simultaneous assays of large numbers of samples.

### 3.3. Divalent Ion Enhancement

Because divalent metal ions have been shown to enhance pre-crRNA processing [76], several studies have tested whether introducing divalent ions can improve the sensitivity of the CRISPR-Cas-based detection system. Extended crRNA with magnesium ions (Mg^2+^) was found to accelerate the activity of Cas12a [70]. When targeting the N gene of SARS-CoV-2, this system, named enhanced analysis of nucleic acids with crRNA extensions (ENHANCE), was found to improve the sensitivity of CRISPR-Cas12a systems up to 23-fold when compared with the unmodified CRISPR-Cas12a-based lateral flow system. MeCas12a, which uses manganese ions (Mn^2+^), was found to increase the sensitivity of SARS-CoV-2 detection [64]. This divalent ion-dependent system enhanced the sensitivity of detection up to 13-fold, enabling the detection of as few as five copies of the SARS-CoV-2 E gene in 1 h 45 min without any special equipment. Although more clinical samples need to be tested, these studies represent a potential strategy for improving the sensitivity of CRISPR-Cas-based molecular detection systems. More rapid and simpler diagnostic methods are important and necessary in pandemic situations. These CRISPR-based diagnostic platforms would likely play key roles under these conditions.

## 4. Discussion and Future Perspectives

Since their original development, CRISPR-Cas systems have been mainly used for genome editing. The ongoing COVID-19 pandemic accelerated advances in CRISPR-Cas-based detection systems for infectious diseases. Compared with the standard RT-PCR method, CRISPR-Cas-based molecular diagnostic methods showed greater sensitivity and target specificity. In addition, CRISPR-Cas-based systems are more time-efficient, less expensive, and easier to perform in the field. Notably, coupling of the unique collateral cleavage activities of Cas12 and Cas13 with various reporter systems has been essential in establishing a wide variety of CRISPR-Cas-based molecular diagnostic platforms. Despite these advantages, CRISPR-Cas-mediated diagnoses of pathogens have several limitations. The nuclease activity of Cas proteins is highly dependent on the specific target sequence, such as G-rich PAM, T-rich PAM, or non-G PFS. The introduction of random mutations into target motifs in virus genomes will prevent pathogen detection. These limitations may be overcome by the simultaneous use of several gRNAs targeting multiple pathogenic regions. In addition, CRISPR-Cas systems have several off-target effects. Careful design of gRNAs using off-target prediction software is required to avoid the recognition of non-specific sequences, as the latter may lead to unexpected false-positive results.

Because the genomes of most infectious viruses undergo rapid mutation, enhancing their ability to infect a broader range of hosts and to survive, future diagnostic tools must be sufficiently flexible to select target regions and capable of simultaneously targeting multiple regions [45]. In addition, further development of simple automated systems can enable the early diagnosis of diseases in large numbers of patients. Artificial intelligence (AI) systems have been introduced recently into the biomedical field. The integration of AI systems with disease diagnosis will enable patients to assess their diagnostic results through cell phone applications. Furthermore, inexpensive portable systems could further contribute to the rapid diagnosis outside of hospitals and in developing countries. CRISPR-Cas-based molecular diagnostic methods may be a promising strategy to prevent potential pandemics.

## Figures and Tables

**Table 1:** Comparison of distinct features of Cas9, Cas12, and Cas13 nucleases.

	Cas9	Cas12	Cas13
Class	2		
Type	II	V	VI
Target	dsDNA	dsDNA, ssDNA	ssRNA
Trans-cleavage activity	N	Y	Y
Target motif	G-rich PAM	T-rich PAM	Non-G PFS
Cleavage pattern	Blunt end	Staggered end	Near U

**Table 2:** Currently available combinations of CRISPR-Cas-mediated diagnostic platforms and reporter systems.

Effect or Enzyme	Reporter System	Diagnostic Platform	Nucleic Acid Extraction	Sample Amplification	Single-Base Resolution	Sensitivity	Turnaround Time	Pathogenic Application	Ref.
Cas12	Fluorescence	DETECTR	N	RPA	N	aM	~2 h	HPV16, HPV18	[8]
		HOLMES	Y	PCR	Y	aM	~3 h	PRV, JEV	[34]
		HOLMESv2	Y	LAMP	Y	nM	~3 h	JEV	[35]
		CDetection	Y	RPA	Y	aM	~2 h	HPV	[29]
		CRISPR-MTB	N	RPA	N	5 copies	~2 h	*Mycobacterium* *tuberculosis*	[36]
		Cas14-DETECTR	N	PCR	N	-	~3 h	-	[10]
	Lateral flow	CRISPR-Cas12a-LFD	N	RAA	N	20 copies	~1 h	ASFV	[37]
		CIA	N	LAMP	N	aM	~1.5 h	*Pseudomonas* *aeruginosa*	[38]
	Fluorescence and lateral flow	Cas12-based POC	N	RPA	N	fM	~3 h	HPV16, HPV18	[39]
		CORDS	Y	RAA	N	aM	~2.5 h	ASFV	[40]
	Electrochemistry	E-CRISPR	N	N/A	Y	pM	~1 h	HPV16, PB-19	[41]
		MAV-chip	N	N/A	Y	-	~1 h	-	[42]
Cas13	Fluorescence	SHERLOCK	Y	RPA	Y	aM	~4 h	ZIKV, DENV	[9]
	Multiplexed fluorescence and lateral flow	SHERLOCKv2	Y	RPA	Y	aM	~3.5 h	ZIKV, DENV	[43]
	Fluorescence and lateral flow	HUDSON +SHERLOCK	N	RPA	Y	aM	~2 h	ZIKV, DENV, WNV, YFV	[44]
	Multiplexed fluorescence	CARMEN-Cas13	Y	PCR/RPA	Y	aM	~3 h	169 Human associated virus	[45]

**Table 3:** CRISPR-Cas-based diagnostic platforms for COVID-19.

Effect or Enzyme	Detection Method	Diagnostic Platform	Nucleic Acid Extraction	Sample Amplification	Single-Base Resolution	Sensitivity	Turnaround Time	Target Gene	Ref.
Cas12	Naked eye	Contamination-free visual Cas12a	Y	RT-LAMP	N	20 copies	~1 h 45 min	ORF	[61]
		CRISPR-Cas12a-NER	Y	RT-RAA	N	10 copies	~1 h 45 min	E	[62]
		CASdetec	Y	RT-RAA	N	10 copies	~2 h	RdRp	[63]
		MeCas12a	Y	RT-RAA	Y	5 copies	~1 h 45 min	E	[64]
		AIOD-CRISPR	Y	NA	N	5 copies	~1 h 40 min	N	[65]
		RT-RPA-CRISPR-Cas12a assay	Y	RT-RPA	N	10 copies	~2 h	S	[66]
		RT-LAMP-Cas12a assay	Y	RT-LAMP	N	30 copies	~1 h 40 min	E, N	[67]
		opvCRISPR	Y	RT-LAMP	N	5 copies	~1 h 45 min	S	[68]
	Fluorescence	CRISPR-FDS	Y	RT-RPA	N	2 copies	~1 h 40 min	ORF1ab, N	[69]
	Lateral flow strip	ENHANCE	Y	RT-LAMP	N	3~300 copies	~1 h 50 min	N	[70]
		SARS-CoV-2 DETECTR	Y	RT-LAMP	N	10 copies	~1 h 40 min	E, N	[71]
	Fluorescence or lateral flow strip	STOPCovid.v2	N	RT-LAMP	N	100 copies	~2 h	N	[72]
	Electrokinetic chip	Microfluidic ITP-CRISPR-based assay	N	RT-LAMP	N	10 copies	~40 min	E, N	[73]
Cas13	Naked eye and later flow strip	SHINE	N	NA	N	>1000 copies	~55 min	ORF1a	[74]
	Fluorescence	CRISPR-COVID	Y	RT-RPA	N	7.5 copies	~1 h 40 min	ORF1ab	[75]
